# Ce-MBGs Loaded with Gentamicin: Characterization and In Vitro Evaluation

**DOI:** 10.3390/jfb14030129

**Published:** 2023-02-26

**Authors:** Francesca Fraulini, Stefano Raimondi, Francesco Candeliere, Raffaella Ranieri, Alfonso Zambon, Gigliola Lusvardi

**Affiliations:** 1 Department of Chemical and Geological Sciences, University of Modena and Reggio Emilia, 41125 Modena, Italy; 2 Department of Life Sciences, University of Modena and Reggio Emilia, 41125 Modena, Italy

**Keywords:** bioactive glasses, cerium, bioactivity, antibacterial activity

## Abstract

Mesoporous Bioactive Glasses (MBGs) are biomaterials widely used in tissue engineering, particularly for hard tissue regeneration. One of the most frequent postoperative complications following a biomaterial surgical implant is a bacterial infection, which usually requires treatment by the systemic administration of drugs (e.g., antibiotics). In order to develop biomaterials with antibiotic properties, we investigated cerium-doped MBGs (Ce-MBGs) as in situ-controlled drug delivery systems (DDSs) of gentamicin (Gen), a wide spectrum antibiotic commonly employed against bacteria responsible of postoperative infections. Here we report the optimization of Gen loading on MBGs and the evaluation of the antibacterial properties and of retention of bioactivity and antioxidant properties of the resulting materials. The Gen loading (up to 7%) was found to be independent from cerium content, and the optimized Gen-loaded Ce-MBGs retain significant bioactivity and antioxidant properties. The antibacterial efficacy was verified up to 10 days of controlled release. These properties make Gen-loaded Ce-MBGs interesting candidates for simultaneous hard tissue regeneration and in situ antibiotic release.

## 1. Introduction

Bioactive glasses are multifunctional materials that have been traditionally adopted in hard tissue engineering as bone fillers, scaffolds and implant coatings, on account of their bioactivity [1,2]. These materials are characterized by the ability to form an apatitic layer in contact with biological fluids, which promotes a stable bond to the living bone [3]. Degradation rate, bioactivity and other specific properties of BGs can be tailored, modifying their composition and morphology, for instance including therapeutic inorganic ions (TTIs) [4,5,6]. The surgical implant of biomaterials often induces a complex inflammatory response correlated to an excessive production of reactive oxygen species (ROS); this condition of oxidative stress can severely aggravate post-operative recovery [7,8]. To restore the redox homeostasis and minimize the total healing time, various antioxidants have been investigated for systemic therapy. However, clinical trials have revealed that systemic treatments can fail to avert ROS-associated diseases and cause severe side effects at high doses [9]. The immunomodulatory response can be regulated, more efficiently exploiting tailored biomaterials that can perform an antioxidant function at the desired location [10]. Cerium has gained interest in this respect given the impressive antioxidant properties of cerium oxide nanoparticles, which derive from the coexistence and reversible interchange between Ce^3+^ and Ce^4+^ oxidation states [11]. A significant amount of research is now involving cerium doping to produce potent antioxidant and anti-inflammatory materials, able to tune the ROS level within the microenvironment [10,12,13]. For instance, we have included cerium into melt-derived BGs [14] and MBGs [15,16], and other researchers have worked on cerium-doped fibers [17], scaffolds [18] and nanoparticles [19]. The antioxidant properties of Ce-BGs are undisputed, but their antibacterial effect is controversial [12]. Since one of the most common complications in bone surgery is postoperative osteomyelitis, in situ antibacterial activity would ensure greater efficacy, lower toxicity and reduced adverse effects compared to a systemic treatment [20]. However, we have recently shown that the antibacterial activity of Ce-BGs is related to the increase in pH which occurs upon BG dissolution, and not to cerium doping, and that the antibacterial effect of BGs is abrogated in buffer media. Ce-BGs are thus likely to be ineffective as antiseptic agents in a homeostatic environment [21]. The loading of Ce-BGs with antibiotics is then a necessary strategy to add an antibacterial effect to the osteogenic and antioxidant properties of Ce-BGs. MBGs have been identified as suitable DDSsfor their high pore volume, specific surface area (SSA) and highly ordered structure. These characteristics allow for more efficient loading and slower and more controllable release kinetics compared to melt-derived or traditional sol–gel-derived BGs [22,23]. Several examples of loading of antibiotics on BGs can be found in the literature: gentamicin [22,24], ampicillin [23], ofloxacin [25], vancomycin [26]. We selected Gen) as a model drug due to its wide spectrum of applications against bacteria causing postoperative infections. Gen has already been included in other bioactive glasses-based systems, such as MBG particles [24], scaffolds [22,27] and fibers [28]. Starting from our optimized Ce-MBGs compositions [15,16], this study investigates the effect of Gen loading on the bioactivity, the antioxidant properties and the antibacterial activity of these Ce-MBGs to obtain multifunctional DDSs. The influence of Ce doping on the loading process is also examined.

## 2. Materials and Methods

### 2.1. MBGs Preparation

As described previously [15,16], MBGs containing different amounts of cerium (0, 1.2, 3.6 and 5.3 mol%) were synthetized by sol–gel evaporation induced self-assembly (EISA) modified method, ground and sieved to produce size of about 250 µm. Cerium percentage was optimized to add beneficial properties without compromising the glassy system properties and the MBGs’ bioactivity [14]. Nominal composition (mol%) of the obtained Ce-MBGs is reported in Table 1. 

### 2.2. Gen Loading

Gen sulphate was purchased from Caesar & Loretz GmbH (Hilden, Germani), with a component distribution (wt%) of 31.7% GenC1, 22.6% GenC1a, 45.7% GenC2, C2a and C2b (Figure S1). The loading solutions were prepared at different Gen concentrations (0.4, 0.8, 1.2, 1.6 and 2.0 mg/mL) in Milli Q water. One gram of each MBG was soaked in 100 mL of loading solution for 8 or 24 h at both 25 °C and 37 °C. We also evaluated whether, in accordance with literature procedures [29,30,31], surface activation of MBGs with acetone before loading to expose the hydroxyl groups, promotes loading. The samples were named “MBGCe_Gen”, according to the amount of cerium in the MBGs and the concentration of the Gen’s solution with Ce = 0, 1.2, 3.6, 5.3 mol% and Gen = 0.4, 0.8, 1.2, 1.6, 2.0 mg/mL.

#### 2.2.1. Gen loading Evaluation

The detection and quantification of Gen on the MBGs were performed by elemental analysis (EA), thermogravimetric analysis (TGA) and specific surface area (SSA) determination. The results are expressed as C/N weight ratio, weight percentage of loaded Gen, hereafter Gen(%), and loading efficiency, hereafter LE(%), calculated as follows, where m = mass:

(1) $$\mathrm{Gen}\left(\%\right)=\frac{m_{\left(loaded\;Gen\right)}}{m_{\left(sample\right)}}\;\times \;100$$

(2) $$\mathrm{LE}\left(\%\right)=\frac{m_{\left(loaded\;Gen\right)}\;}{m_{\left(Gen\;in\;loading\;solution\right)}}\;\times \;100$$

EA was performed with a Thermo Scientific Flash 2000 Organic Elemental Analyzer. TGA was carried out in a Seiko SSC 5200 Analyzer (Hitachi, Tokyo, Japan)) using an airflow of 100 µL/min and heating from 25 to 800 °C at 10 °C/min. SSA was determined by nitrogen adsorption porosimetry using a Micromeritics Chemisorb 2750 and the Brunauer–Emmett–Teller (BET) method [32]. 

Gen(%) was also determined by high-performance liquid chromatography (HPLC) for MBG5.3_0.8 only by measuring the Gen concentration in the loading solution before and after loading. HPLC analysis was performed by an Infinity 1120 apparatus (Agilent Technologies, Santa Clara, United States) equipped with a quaternary pump and an evaporative light scattering detector. The elution was carried out at 60 °C on a ZORBAX SB-C18 (4.6 mm × 150 mm, 3.5 µ) (Agilent Technologies, Santa Clara, United States), with 0.2 mol/L TFA in water containing 8% methanol and a flow rate of 1.0 mL/min. The detector was set at 3.5 bar, gain, and filter 5s. Twenty microliters of sample volume were injected.

After loading, the morphology and the maintenance of the mesoporous structure of MBGs were evaluated by Scanning Electron Microscopy (SEM) and Transmission Electron Microscopy (TEM). Analyses were carried out with a JEOL JSM-6010LA microscope (equipped with Electron Dispersive Spectroscopy, EDS, Leica Microsystems Wetzlar, Germany) and a TEM-FEG Talos F200W G2 microscope (Thermo Fischer, Waltham, MA, USA), respectively. 

Following previous papers [24], a UV–Vis determination was attempted. Results are not reported since they were not reproducible. 

#### 2.2.2. Gen Release Profile

On the most promising samples, the Gen release profile in simulated body fluid (SBF) was evaluated by TGA and HPLC analyses. The measurements were carried out in SBF in order to simulate the physiological environment in which these materials should be implanted. For TGA, the Gen release was estimated by the difference between the weight loss in the 200–400 °C range, assigned to the loss% of Gen. All TGA data were corrected for the baseline values. For HPLC, the release was evaluated by Gen concentration in the solution after SBF loading.

### 2.3. In Vitro Bioactivity Assessment

To evaluate the retention of bioactivity, 1.5 mg of MBGCe_Gen was soaked in 1 mL of SBF at 37 °C for 1, 2, 3, 4, 6, 7, 9 and 10 days to verify the formation of an apatitic layer constituted of hydroxyapatite (HA) [33,34]. 

After SBF soaking, the samples were characterized by X-Ray Powder Diffraction (XRPD) with an X’Pert PRO-PANAnalytical diffractometer (Malvern Panalytical, Malvern, United Kingdom)) to verify the presence of crystalline HA (hydroxyapatite, Ca_10_(PO_4_)_6_(OH)_2_), Scanning Electron Microscopy (SEM/EDS) to evaluate morphological changes and to estimate the molar ratio Ca/P and Fourier transform infrared (FTIR) spectroscopy with an FTIR Perkin Elmer (Waltham, Massachusetts, United States) 160 spectrometer to verify the presence of characteristic bands of HA.

### 2.4. Antioxidant Activity Assay

The antioxidant property of MBGCe_Gens was estimated as their ability to remove H_2_O_2_, one of the most significant ROS species. In correlation with the role of enzyme Catalase, the property is named CAT activity. The tests were performed using the Fluorimetric Hydrogen Peroxide Assay Kit from Sigma Aldrich with a TECAN GeniosPro microplate reader, as presented in a previous paper [16] Again, measurements were performed in SBF to simulate the physiological environment surrounding the implant. The presence of H_2_O_2_ in SBF is detected through its reaction with a molecular probe catalyzed by the peroxidase enzyme, which generates a red fluorescent product that can be analyzed fluorometrically. CAT activity is reported as the percentage of H_2_O_2_ decomposed at the end of the assay. We suspended 40 mg of MBGCe_Gen in 400 μL of 50 μM solution of H_2_O_2_ in SBF and measured the residual concentration of H_2_O_2_ after 120 min of soaking.

### 2.5. Antibacterial Tests

The antibacterial activity of MBGCe_Gen was investigated by an adaptation of the Kirby–Bauer agar diffusion method. Briefly, 10 mg of each sample was deposited in a sole spot over a plate filled with 15 mL of Luria-Bertani agar (LBA; 5 g/L yeast extract, 10 g/L tryptone, 10 g/L NaCl, 15 g/L agar). A disk containing 10 mg of Gen was placed on each plate as the inhibition reference. Five milliliters of liquid LBA, kept at 45 °C, was seeded with a fresh overnight culture of *Escherichia coli* ATCC11229 to obtain a concentration of approx. 10^6^ cfu/mL, and carefully poured over the plate. The growth inhibition was verified after overnight incubation at 37 °C by measuring the diameters of the inhibition halos. The tests were performed comparing MBGs undoped and doped with 5.3 mol% of cerium, unloaded and loaded with Gen (0.4, 0.8, 1.2 mg/mL) before and after SBF soaking (1–10 days). We verified the retention of the antibacterial properties of simulated implantation by analyzing the materials after 10 days of SBF soaking. In order to assess a dose–response effect of the MBG loaded with Gen, the inhibition halos on agar plates were measured by depositing the same amount of glass powder (10 mg), but increasing amounts of Gen (0–500µg). The different glass spots were obtained by mixing various ratios of the unloaded MBG with the MBG0.8Gen.

## 3. Results and Discussion

To identify MBGCe_Gen with optimal and persistent bioactivity and antioxidant properties, we first studied the Gen loading process by EA, TGA, SSA, SEM and TEM analyses on MBGs with different cerium content. 

Once MBGs with optimized cerium and Gen content were identified, we profiled their bioactivity by XRPD, FTIR and SEM/EDS techniques; the bioactivity tests (SBF)TGA and HPLC analyses allowed us to monitor Gen release. 

Antioxidant activity in SBF has been tested by CAT enzymatic assay. 

Finally, we evaluated their antibiotic properties and Gen release behavior in SBF by an adaptation of the Kirby–Bauer agar diffusion method.

### 3.1. Gen Loading Evaluation

#### 3.1.1. Elemental Analysis (EA)

In preliminary experiments, we evaluated the effect of surface activation, temperature and soaking time on the loading process of Ce-MBGs. The Gen loaded amount did not change appreciably when activating the surface, nor when using a loading temperature of 37 instead of 25 °C and a contact time of 8 instead of 24 h. We then chose 24 h, 25 °C, no surface activation as standard loading conditions. 

Determining the content of C and N by EA allows to assess the amount of Gen loaded on the Ce-MBGs. In all cases, we obtained a C/N weight ratio between 3.0 and 3.7, in line with the Gen theorical value of 3.4. (Table S1). Interestingly, all samples were S-free, indicating that Gen is not loaded as sulfate salt. 

**Figure 1:**
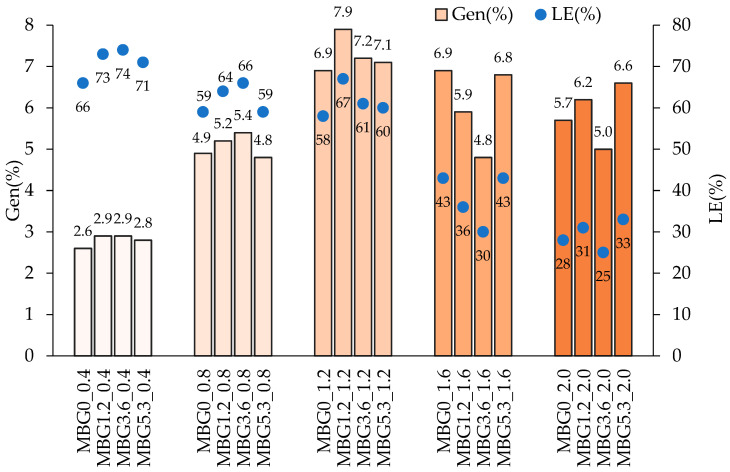
Gen(%) and LE(%) for Ce-MBGs at different concentrations of Gen loading solution.

Figure 1 shows the effect of the Gen loading solution concentration (0.4–2.0 mg/mL) on the Gen loaded amount, expressed as Gen(%) and LE(%) (Table S1).

In all cases, the amount of cerium did not significantly affect the Gen(%) nor LE(%) of the final materials; it is therefore possible to take full advantage of both Ce doping and Gen loading independently, in order to identify MBGs with optimal antioxidant and antibacterial properties and with persistent bioactivity

Interestingly, Gen(%) increased with the concentration of the loading solution up to 1.2 mg/mL and remained constant at higher concentrations. Gen(%) was around 3, 5 and 7% for Gen = 0.4, 0.8 and 1.2 mg/mL respectively, but loading with Gen = 1.6 and 2.0 mg/mL still resulted in a Gen(%) of 7% or less. Correspondingly, LE(%) was in the range 58–74% up to a concentration of 1.2 mg/mL, and decreased to 25–43% at higher loading concentrations, as the loading content remained constant but the loading solutions were more concentrated. 

Ce-MBGs loaded with 0.8 and 1.2 mg/mL of Gen are therefore the best possible candidates for further evaluating bioactivity and antioxidant and antibacterial properties, as they have the largest amount of loaded Gen and the most efficient loading processes. 

In Section 3.2 we will show that Ce-MBGs loaded with 0.8 mg/mL of Gen retain excellent bioactivity, which is, in contrast, severely hampered by loading with 1.2 mg/mL of Gen; therefore, we identified the Ce-MBGs loaded with 0.8 mg/mL as the most promising materials.

#### 3.1.2. Thermogravimetric Analysis (TGA)

TGA analyses were performed on MBG with the highest cerium amount (MBG5.3) after loading with 0.4, 0.8 and 1.2 mg/mL to assess the mass loss as the loaded Gen increased. 

For comparison purposes, TGA was also performed on the starting Gen sulfate reagent. The TGA of Gen sulfate reveals three weight losses (WL): 25–200 °C (WL1), 200–400 °C (WL2), 400–800 °C (WL3), as shown in Table 2.

**Figure 2:**
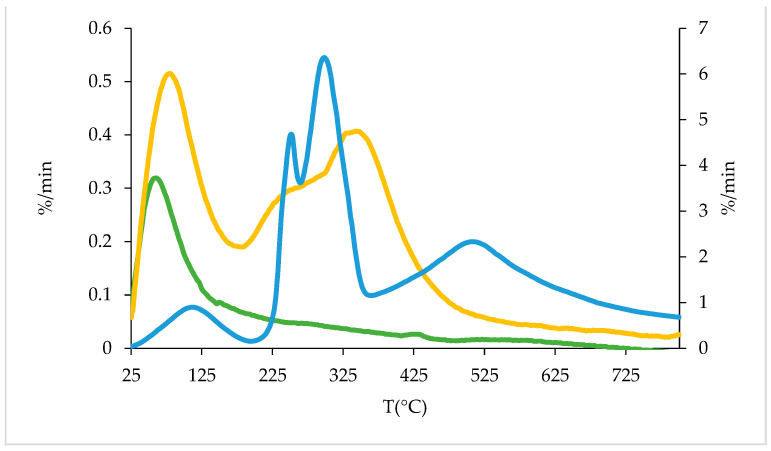
DTG profiles for Gen sulfate (blue), MBG5.3 (green) and MBG5.3_0.8 (yellow).

Unloaded MBG5.3 showed only WL1, which can be attributed to water loss, while loaded MBG5.3 showed a weight loss in the 200–400 °C range (WL2), attributed to Gen loss. A weight loss between 400–800 °C (WL3) was observed in the Gen sulfate only, as shown by the DTG curves in Figure 2, and it corresponds to the sulfate ion decomposition. This confirms that Gen is loaded without its sulfate counterion, as suggested by EA analysis (paragraph 3.1.1 and Table S1). Furthermore, the percentages obtained are in line with the values reported in Figure 1. 

#### 3.1.3. Specific Surface Area (SSA) Determination

**Figure 3:**
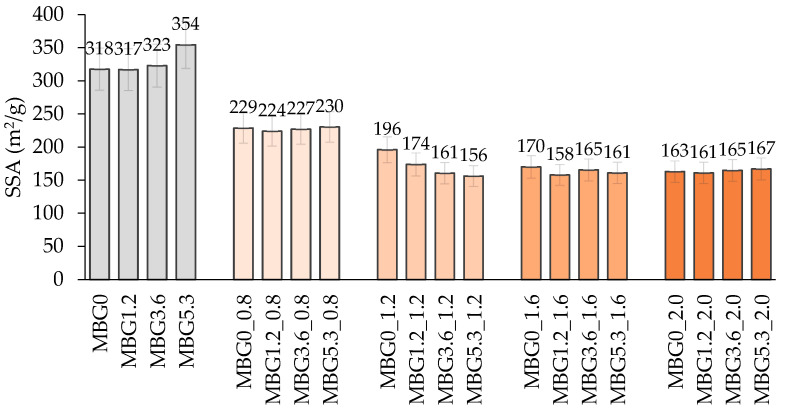
SSA (m^2^/g) of Ce-MBGs before and after Gen loading.

The SSA values of the unloaded Ce-MBGs (Figure 3) showed no significant dependence on the amount of cerium and all samples had SSA values above 300 m^2^/g, in accordance with their mesoporous structure [23]. Upon loading with Gen, the SSA values decreased significantly to 156–230 m^2^/g, suggesting a material with a certain degree of porosity. Again, no correlation between the SSA of the loaded materials and the amount of cerium in Ce-MBGs was observed, as already verified by Gen(%) (Figure 1). The trend of Figure 3 suggests a correlation between SSA decrease and Gen(%) increase, as we can reasonably ascribe the decrease in SSA to the pore occlusion that occurs during Gen loading; accordingly, MBGs_08 showed higher SSA than MBGs_1.2, MBGs_1.6 and MBGs_2.0 that present higher Gen(%).

#### 3.1.4. Morphological Evaluation (SEM, TEM)

**Figure 4:**
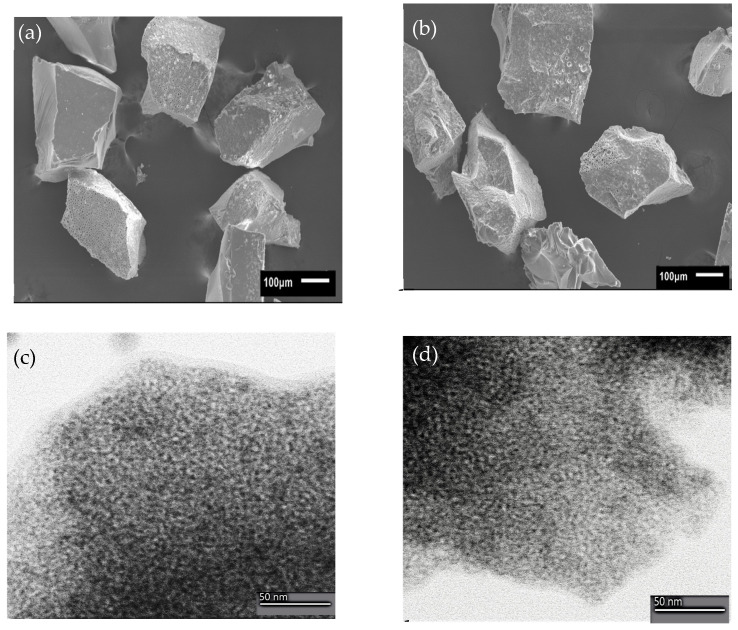
SEM micrographs of MBG5.3 (**a**) and MBG5.3_0.8 (**b**). TEM micrographs of MBG5.3 (**c**) and MBG5.3_0.8 (**d**).

SEM and TEM analyses (Figure 4) were performed on both unloaded and loaded Ce-MBGs to observe the morphological changes after Gen loading. The evaluation was carried out on the most promising loaded samples (Gen = 0.8 mg/mL), as mentioned in Paragraph 3.1.1. SEM micrographs (Figure 4a,b) revealed that the morphology of Ce-MBGs was not altered by Gen loading. TEM micrographs confirm the preservation of the characteristic mesoporous structure (Figure 4c,d). The presence of cerium did not influence this behavior. 

### 3.2. In Vitro Bioactivity Evaluation 

After having evaluated the Gen loading on the Ce-MBGs, we investigated the bioactivity on the most promising MBGCe_Gen (Gen = 0.8 and 1.2 mg/mL), the samples with the highest Gen(%) at the maximum LE(%). 

**Figure 5:**
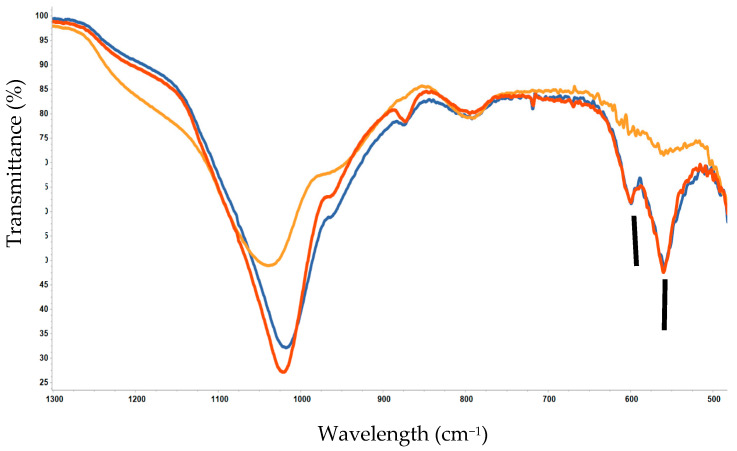
FTIR spectra of MBG5.3 (red), MBG5.3_0.8 (blue) and MBG5.3_1.2 (yellow) after 3 days in SBF.

FTIR studies showed that the formation of the apatitic layer for MBGs_1.2 was delayed compared to MBGs_0.8 (Figure 5); in fact, the characteristic bands of HA were less solved. Specifically, these bands are identified near 605 and 565 cm^−1^ (indicated with vertical bars) and attributed to the IR-active 𝜈_4_ deformation mode of PO_4_^3-^ ions of Td symmetry [35,36]. 

However, no significant differences were observed between unloaded MBG5.3 and MBG5.3_0.8, as reported in Figure 5. Gen loading at 0.8 mg/mL did not seem to affect the bioactivity of the Ce-MBGs and was thus chosen for further evaluation. The bioactivity evaluation was carried out after 1–10 days of soaking in SBF; Figure 5 shows the Ce-MBG with highest Ce percentage (5.3 mol%) after 3 days of soaking in SBF, which is the minimum time for the formation of a quite crystalline apatitic layer. 

**Figure 6:**
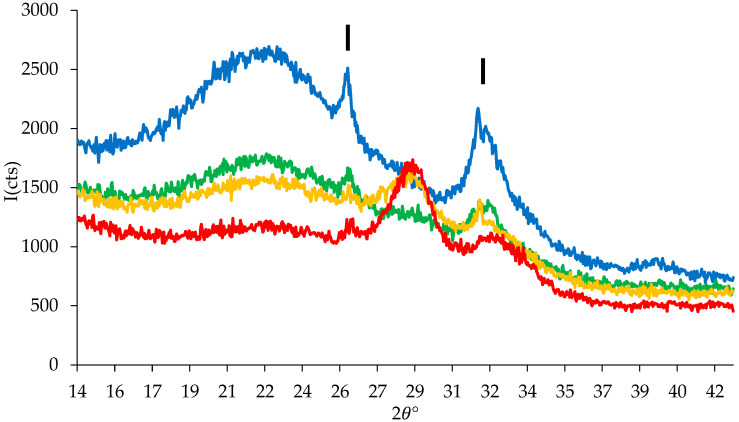
XRPD patterns of MBG (blue), MBG1.2 (green), MBG3.6 (yellow) and MBG5.3 (red) loaded with 0.8 mg/mL Gen solution after 3 days in SBF.

All XRPD patterns (Figure 6) showed characteristic peaks associated to HA interplanar distances of 3.46 Å (2𝜃° = 26.0) and 2.81 Å (2𝜃° = 31.9), indicated with vertical bars. At higher cerium content (3.6 and 5.3%), a peak at 3.09 Å (2𝜃° = 29.2) belonging to cerium phosphate was also present, in agreement with our previous studies [15].

**Figure 7:**
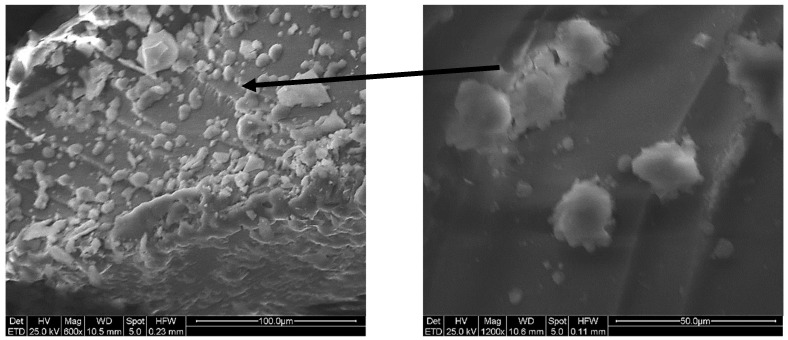
SEM micrographs of MBG5.3_0.8 after 3 days in SBF at low (left) and high magnification (right).

The formation of HA aggregates of spherical shape on the MBG5.3_0.8 surface was also detected by SEM (Figure 7). The right panel highlights a detail of the spherical agglomerates, with Ca/P molar ratio of ~1.6, from EDS analysis, not far from the stochiometric value of 1.67 for HA.

### 3.3. Gen Release Evaluation

#### 3.3.1. Thermogravimetric Analysis (TGA) after SBF Soaking

TGA analysis was performed on MBG5.3_0.8 soaked in SBF for 1, 2 and 3 days to monitor the release of Gen (Table 3). Pleasingly, the results showed WL2 and WL3 quite close to those of the MBG5.3_0.8 before SBF soaking. These results suggested a very slow Gen release, with a ~15% loss of total loaded Gen within the first 24 h and a slower release at longer times. 

#### 3.3.2. High-Performance Liquid Chromatography (HPLC) after SBF Soaking

Gen(%) for MBG5.3_0.8 was measured by HPLC; the resulting value of 5.3% is in good agreement with the EA analysis for the same sample. 

**Figure 8:**
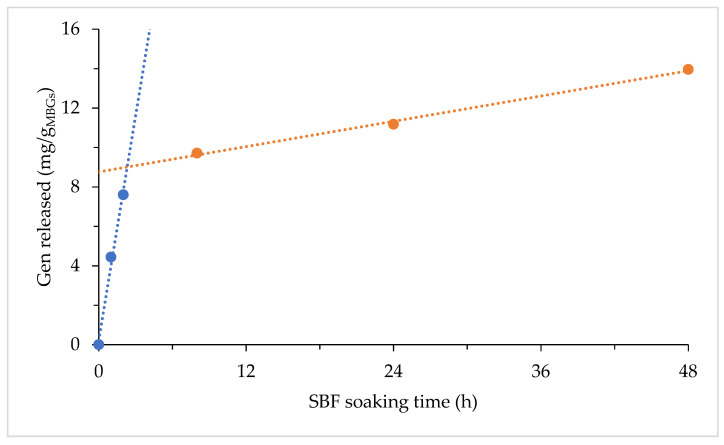
Gen release for MBG5.3_0.8 after SBF soaking.

HLPC was performed on MBG5.3_0.8 at varying immersion times in SBF to evaluate Gen release. The results reported in Figure 8 suggest two distinct release kinetics, with a fast Gen loss occurring in the first 3 h followed by a much slower one at longer times. Gen loss after 1 day is 11 mg per gram of MGB5.3_08, corresponding to ~20% loss of the total loaded Gen. This result is again in good agreement with that obtained by TGA.

### 3.4. Antioxidant Properties

**Figure 9:**
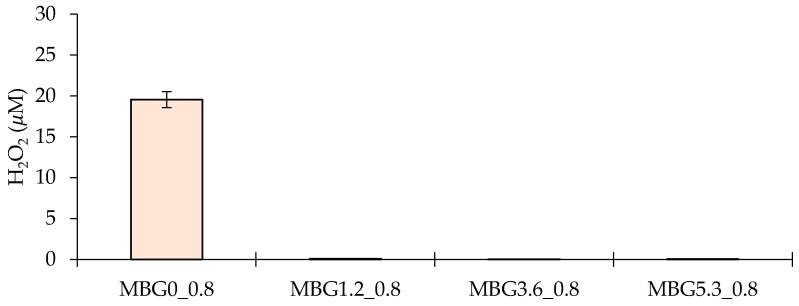
Residual H_2_O_2_ concentrations (µM) after 120 min of contact.

The CAT activity of MBGs_0.8 was tested measuring the residual H_2_O_2_ concentration after 120 min of contact between 40 mg of MBGs_0.8 and 400 µL of 50 µM solution of H_2_O_2_ in SBF (Figure 9).

As previously observed [16], only cerium-doped MBGs showed CAT-like activity, and the high SSA of the mesoporous structures increased their ability to dismutate H_2_O_2_. Figure 9 shows that all residual H_2_O_2_ concentrations were below 1 µM in Ce-doped MBGs, regardless of Gen loading. Once again, it is confirmed that it is possible to take full advantage of both Ce and Gen addition to the MBGs.

### 3.5. Antibacterial Tests

**Figure 10:**
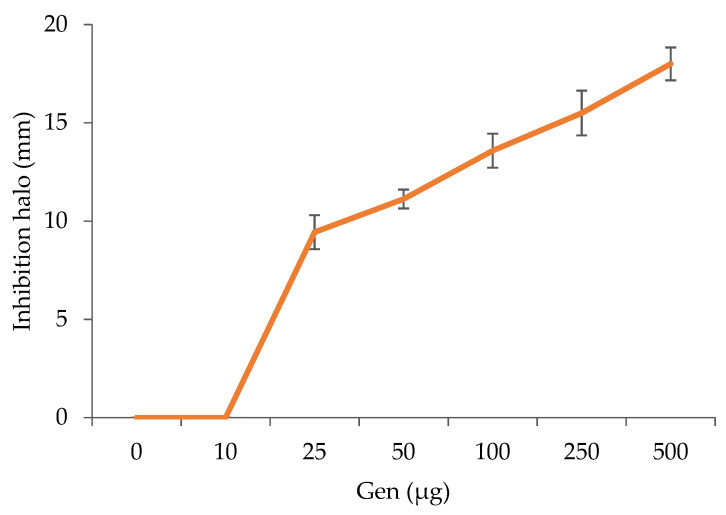
*E. coli* ATCC11229 growth inhibition: dose–response effect of the MBG loaded with Gen. The observed halos were obtained by depositing the same amount of glass powder (10 mg) but increasing amounts of Gen (0–500 µg). The different glass spots contained the unloaded MBG and the MBG0.8 Gen mixed in various ratios. The inhibition halo diameters were reported as the mean value (mm) of at least three replicate experiments.

The antibacterial activity of loaded and unloaded MBGs is reported in Figure S2. Independently from the amount of cerium, unloaded MBGs did not show any antibacterial activity: no inhibition halos were observed in absence of Gen loading (Figure S2a). This evidence is in agreement with our previous results, which linked the reported antibacterial activity of Ce-MBGs to the pH increase induced by their dissolution [13]. On the other hand, Gen loading conferred to MBGs a clear inhibitory activity towards *E. coli* growth (Figure S2). When varying the amount of Gen deposited on agar dishes from 10 to 500 µg, MBGCe_Gen showed halos with diameters ranging from 0 to 18 mm, with no growth inhibition observed at the lower amount (Figure 10), suggesting a sequestration effect on small amounts of antibiotic that, on the contrary, was likely less strongly bound when loaded in a higher amount.

**Figure 11:**
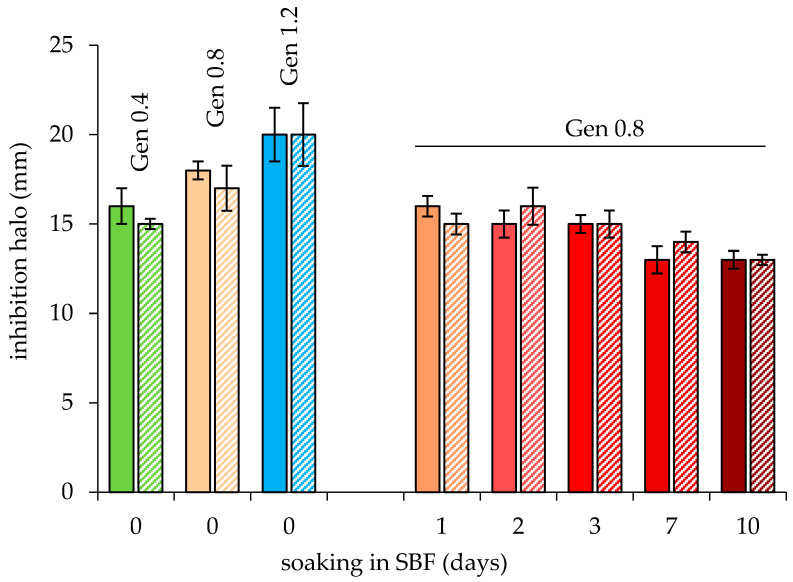
Evaluation of the inhibitory effect of MBG-Gen on the growth of *E. coli* ATCC11229, comparing glass prepared with increasing concentrations of Gen in the loading solution. For MBG-0.8Gen, the effect of soaking in SBF was also assessed. The inhibition halo diameters were reported as the mean value (mm) of at least three replicate experiments. MBG (full line), MBG5.3 (diagonal line).

Coherently, the inhibition effect grew when glasses were prepared with loading solutions at increasing concentrations of Gen (1.2 ≥ 0.8 > 0.4; Figure 11). As concluded in the previous sections, the most promising samples are MBGs_0.8, retaining a fast bioactivity combined with the excellent antioxidant properties of the cerium-containing samples. Pleasingly, these samples showed inhibition halos that did not significantly differ from the MBG_1.2 ones. Interestingly, prolonged soaking in SBF of the loaded MBGs caused a decrease of the antibacterial activity in the first 24 h. After the first day, the antibacterial activity was retained up to day 3 and decreased gradually afterwards; at day 10, the inhibition halos are still persisting. This is in accordance with the release of Gen observed by TGA (Table 3), in which Gen loss at 24 h was around 15% but then was undetectable for up to 3 days. This behavior is consistent with the presence on the MBGs of strongly bound Gens and some weakly bound ones. During the first hours of simulated exposure to biological fluids, it is likely that the weakly bound Gen is released relatively quickly, explaining the marked antibacterial activity of loaded glasses. Then, the strongly bound Gen is released with a slower kinetic compatibly with the prolonged antibacterial action observed up to the tenth day of soaking. A similar behavior was described by Arcos et al. [37] for glass/PMMA composites characterized by a fast release of the antibiotic during 15 h of soaking, followed by a slower release stage, resulting in values of 90% of released Gen after 14 days. 

## 4. Conclusions

We evaluated the antibacterial properties, retention of bioactivity and antioxidant properties of several Ce-MBGs loaded with Gen solutions with concentrations ranging between 0.4 and 2.0 mg/mL. 

Quantification of loading showed a Gen(%) around 7% with a LE(%) of 60% up to 1.2 mg/mL loading solution concentration, independent of cerium amount. 

Loading solutions at higher concentrations did not affect Gen(%) but decreased the LE(%).Ce-MGBs loaded with a 0.8 mg/mL Gen solution showed good bioactivity and retained the antioxidant properties of the parent Ce-MBG, while the bioactivity was severely hampered when loading was performed at higher 1.2 mg/mL concentration. For these reasons, loading with 0.8 mg/mL was chosen as the most promising process, allowing us to take the maximum advantage of both Ce doping and Gen loading.

When soaked in SBF, the loaded MBGs displayed a distinct release kinetics, with a 15% loss of total loaded Gen over the first 24 h, then a slow release over 10 days. 

This behavior translated into a retained antibacterial activity, with visible halos of inhibition up to 10 days. 

This sustained release could represent a fundamental feature of Gen-loaded Ce-MBGs for their efficacy in tissue regeneration, contributing to the reduction of infection risk and the bloom of inflammation after implant.

## Figures and Tables

**Table 1:** Nominal composition (mol%) of Ce-MBGs.

MBGs	SiO_2_	CaO	P_2_O_5_	CeO_2_
MBG0	80.0	15.0	5.0	0
MBG1.2	79.1	14.8	4.9	1.2
MBG3.6	77.1	14.5	4.8	3.6
MBG5.3	75.8	14.2	4.7	5.3

**Table 2:** Weight loss, expressed as WL(%), for different temperature ranges.

	WL1(%) 25–200 °C	WL2(%) 200–400 °C	WL3(%) 400–800 °C	Total WL(%)
Gen sulfate	8.4	47.7	43.9	100
MBG5.3	3.0	0.8	0.3	4.1
MBG5.3_0.4	8.2	4.9	0.7	13.8
MBG5.3_0.8	5.4	7.8	1.2	14.4
MBG5.3_1.2	6.8	8.7	1.7	17.2

**Table 3:** Weight loss WL (%) for different temperature ranges.

	WL1(%) 25–200 °C	WL2(%) 200–400 °C	WL3(%) 400–800 °C	Total WL(%)
MBG5.3_0.8	4.9	7.1	1.2	13.2
MBG5.3_0.8 SBF 1d	4.3	6.0	1.1	11.4
MBG5.3_0.8 SBF 2d	3.5	6.2	1.2	10.9
MBG5.3_0.8 SBF 3d	4.2	5.8	1.2	11.2

## Supplementary Materials

The following supporting information can be downloaded at: https://www.mdpi.com/article/10.3390/jfb14030129/s1, Figure S1. Structure of gentamicin’s different components; Figure S2. Antibacterial test with an adaptation of the agar diffusion method. (a) MBGs undoped (I) and doped with 5.3 mol% of cerium (II); a control disk containing Gen was placed at the center of the plate. No inhibition halos were observed in absence of Gen. (b) MBGs loaded with Gen (0.4, 0.8,1.2 mg/mL); Table S1. Weight C/N ratio for Ce-MBGs at different Gen concentrations; Figure S2. Gen(%) and LE(%) for Ce-MBGs at different concentrations of Gen loading solution.
