# Supplementary material for: Ce-MBGs Loaded with Gentamicin: Characterization and In Vitro Evaluation
Source: J Funct Biomater. 2023 Feb 26;14(3):129. doi: 10.3390/jfb14030129 (PMC10054597; doi:10.3390/jfb14030129)
Supplement: Supplementary file 1 [file jfb-14-00129-s001.zip › jfb-2195417-supplementary.pdf]

Supplementary Material

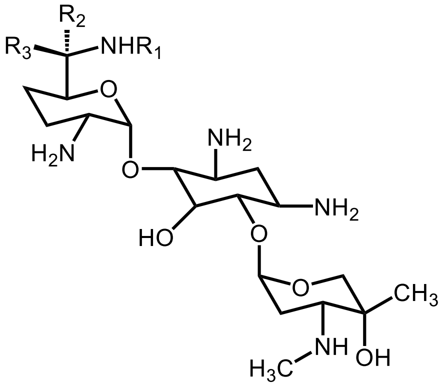

Component	R_1	R_2	R_3
**C1**	CH_3	H	CH_3
**C1a**	H	H	H
**C2**	H	H	CH_3
**C2a**	H	CH_3	H
**C2b**	CH_3	H	H

Figure S1. Structure of gentamicin's different components.

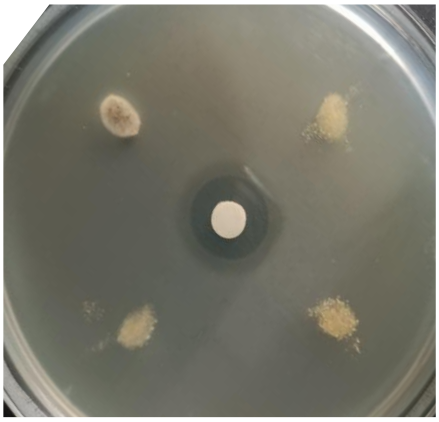

a)

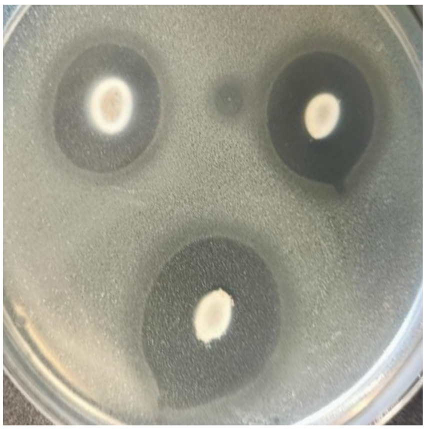

b)

Figure S2. Antibacterial test with an adaptation of the agar diffusion method. (**a**) MBGs undoped (I) and doped with 5.3 mol% of cerium (II); a control disk containing Gen was placed at the centre of the plate. No inhibition halos were observed in absence of Gen. (**b**) MBGs loaded with Gen (0.4, 0.8,1.2 mg/mL).

Table S1. Weight C/N ratio for Ce-MBGs at different Gen concentrations.

	C/N
**MBG0_0.4**	3.7
**MBG1.2_0.4**	3.0
**MBG3.6_0.4**	3.7
**MBG5.3_0.4**	3.0
**MBG0_0.8**	3.5
**MBG1.2_0.8**	3.5
**MBG3.6_0.8**	3.5
**MBG5.3_0.8**	3.6
**MBG0_1.2**	3.6
**MBG1.2_1.2**	3.7
**MBG3.6_1.2**	3.6
**MBG5.3_1.2**	3.6
**MBG0_1.6**	3.5
**MBG1.2_1.6**	3.6
**MBG3.6_1.6**	3.6
**MBG5.3_1.6**	3.7
**MBG0_2.0**	3.6
**MBG1.2_2.0**	3.3
**MBG3.6_2.0**	3.2
**MBG5.3_2.0**	3.5

Table S2. Gen(%) and LE(%) for Ce-MBGs at different concentrations of Gen loading solution.

Gen[mg/mL]	MBGs	Gen(%)	LE(%)
**0.4**	**MBG0_0.4**	2.6	66
	**MBG1.2_0.4**	2.9	73
	**MBG3.6_0.4**	2.9	74
	**MBG5.3_0.4**	2.8	71
**0.8**	**MBG0_0.8**	4.9	59
	**MBG1.2_0.8**	5.2	64
	**MBG3.6_0.8**	5.4	66
	**MBG5.3_0.8**	4.8	59
**1.2**	**MBG0_1.2**	6.9	58
	**MBG1.2_1.2**	7.9	67
	**MBG3.6_1.2**	7.2	61
	**MBG5.3_1.2**	7.1	60
**1.6**	**MBG0_1.6**	6.9	43
	**MBG1.2_1.6**	5.9	36
	**MBG3.6_1.6**	4.8	30
	**MBG5.3_1.6**	6.8	43
**2.0**	**MBG0_2.0**	5.7	28
	**MBG1.2_2.0**	6.2	31
	**MBG3.6_2.0**	5.0	25
	**MBG5.3_2.0**	6.6	33
